# System degeneration in an MM1-type sporadic Creutzfeldt-Jakob disease case with an unusually prolonged akinetic mutism state

**DOI:** 10.1080/19336896.2020.1868931

**Published:** 2021-01-20

**Authors:** Yasushi Iwasaki, Keiko Mori, Masumi Ito, Yoshinari Kawai, Akio Akagi, Yuichi Riku, Hiroaki Miyahara, Atsushi Kobayashi, Tetsuyuki Kitamoto, Mari Yoshida

**Affiliations:** a Department of Neuropathology, Institute for Medical Science of Aging, Aichi Medical University, Nagakute, Japan; b Department of Neurology, Oyamada Memorial Spa Hospital, Yokkaichi, Japan; c Laboratory of Comparative Pathology, Faculty of Veterinary Medicine, Hokkaido University, Sapporo, Japan; d Department of Neurological Science, Tohoku University Graduate School of Medicine, Sendai, Japan

**Keywords:** Creutzfeldt-Jakob disease, akinetic mutism state, panencephalopathic-type, pyramidal tract degeneration, system degeneration

## Abstract

Methionine/methionine type 1 (MM1-type) sporadic Creutzfeldt-Jakob disease (sCJD), known as the ‘classic type,’ shows typical clinicopathological sCJD findings. In general, patients reach an akinetic mutism state within a few months of disease onset and die soon after if supportive therapies are not administered. Here, we describe remarkable neuropathologic observations of MM1-type sCJD in a 48-year-old, Japanese man with an unusually prolonged akinetic mutism state. In the early disease stages, the patient exhibited abnormal behaviour with gait disturbance and rapidly progressive cognitive dysfunction. Diffusion-weighted magnetic resonance imaging revealed extensive cerebral cortical hyperintensity. Prion protein (PrP) gene analysis revealed no mutations, and the polymorphic codon 129 exhibited methionine homozygosity. Although the patient remained stable with tube feeding for more than 2 years after reaching the akinetic mutism state, he died because of central respiratory failure 30 months after disease onset. Neuropathologic investigation showed extensive devastating lesions, such as status spongiosus, and typical spongiform changes could no longer be observed in the cerebral neocortex. Conspicuous pyramidal tract degeneration was observed. However, the regions commonly preserved in MM1-type sCJD pathology were still relatively preserved. Immunostaining revealed extensive diffuse synaptic-type PrP deposition in the grey matter. The pathological findings suggested that sCJD is a neurodegenerative disease that shows system degeneration; there are primary and secondary degenerative regions and distinct preserved regions, even in cases with prolonged disease duration. In addition, it is considered that there is a limited survival period for MM1-type sCJD, even if active symptomatic treatment is provided.

## Introduction

The clinical and pathological presentations of sporadic Creutzfeldt-Jakob disease (sCJD) are strongly influenced by the polymorphism at codon 129 of the prion protein (PrP) gene (methionine [Met] or valine [Val]; Met/Met, Met/Val, or Val/Val) and the type of protease-resistant PrP (type 1 PrP^Sc^ or type 2 PrP^Sc^) [1,2]. Therefore, to accurately classify and understand each sCJD case, comprehensive analyses of clinical and neuropathological findings as well as analyses of the PrP gene and PrP^Sc^ type are needed [1,2]. Based on these analyses’ results, sCJD can be classified into six subtypes: MM1, MM2, MV1, MV2, VV1, and VV2 [1]. Methionine/methionine type 1 (MM1-type) is the most common type and represents the typical clinical features of sCJD, namely, myoclonus, periodic sharp-wave complexes (PSWCs) on electroencephalography (EEG), and progressive cognitive impairment; therefore, this type referred to as the ‘classic type’ [1,2]. In general, the disease progression of MM1-type sCJD is very rapid; patients with MM1-type sCJD reach an akinetic mutism state within several months of disease onset, and they die shortly after when symptomatic treatment, such as a tube feeding, is not provided [1,2].

The severity of sCJD pathology shows varying associations not only with disease duration and each sCJD subtype but also in each neuroanatomical region, and the affected regions generally tend to incur progressive destructive changes along with disease prolongation [1–6]. However, it is not well understood how the degeneration of each region is associated with disease prolongation. Here, we report the clinical and neuropathological findings of a patient with MM1-type sCJD who had a prolonged akinetic mutism state with subsequent tube feeding. Further, we discuss the neuropathological aspects with respect to the progression and system degeneration of sCJD pathology. To the best of our knowledge, of the previously reported autopsy cases of MM1-type sCJD with a classic clinical presentation, this case had the longest akinetic mutism state.

## Results

### Macroscopic findings

**Figure 1.**
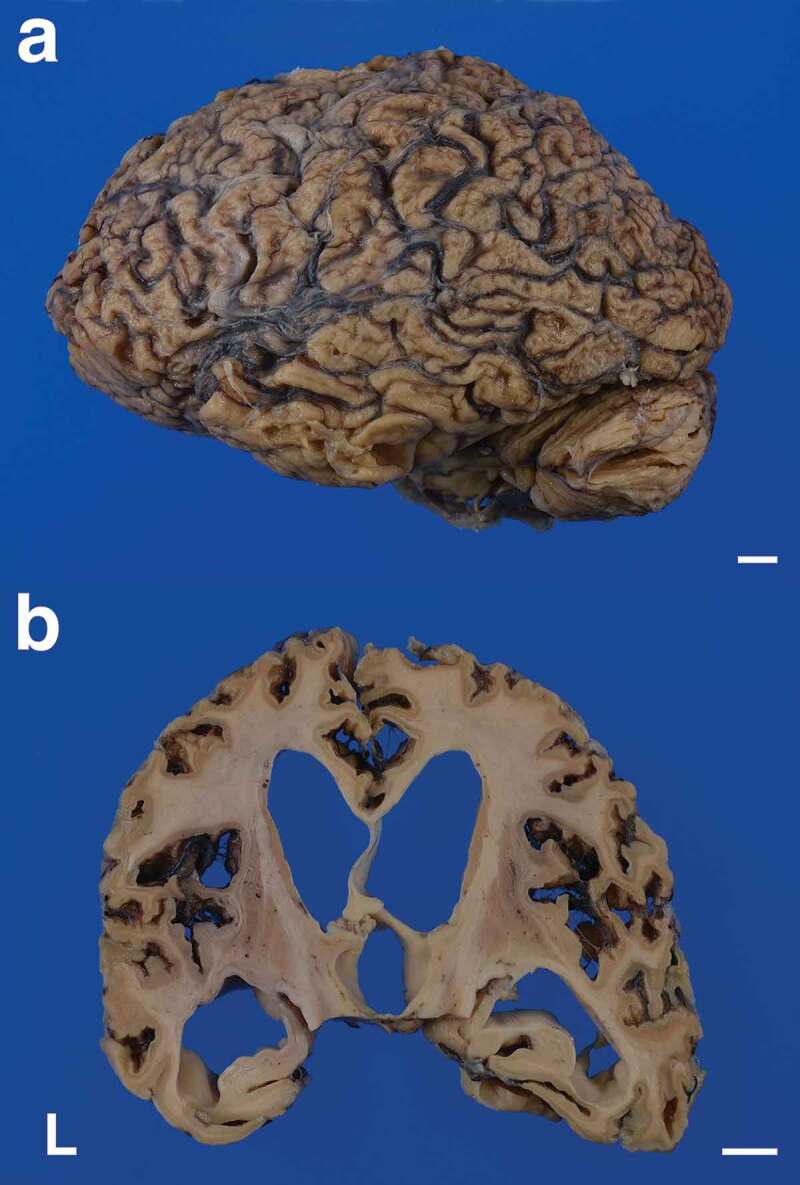
Macroscopic appearances of the formalin-fixed brain. (a) Severe cerebral atrophy with widening of the sulci is recognized from the frontal to the occipital lobes. The cerebellum also shows severe atrophy. (b) Coronal section of the cerebrum shows severe thinning of the neocortex. Severe enlargement of the lateral and third ventricles is also apparent, but the hippocampal formation is relatively preserved from atrophy. The striatum, medial thalamus, and white matter also show atrophy. The corpus callosum is extremely thin. Scale bars: 10 mm. L, left side

**Figure 2.**
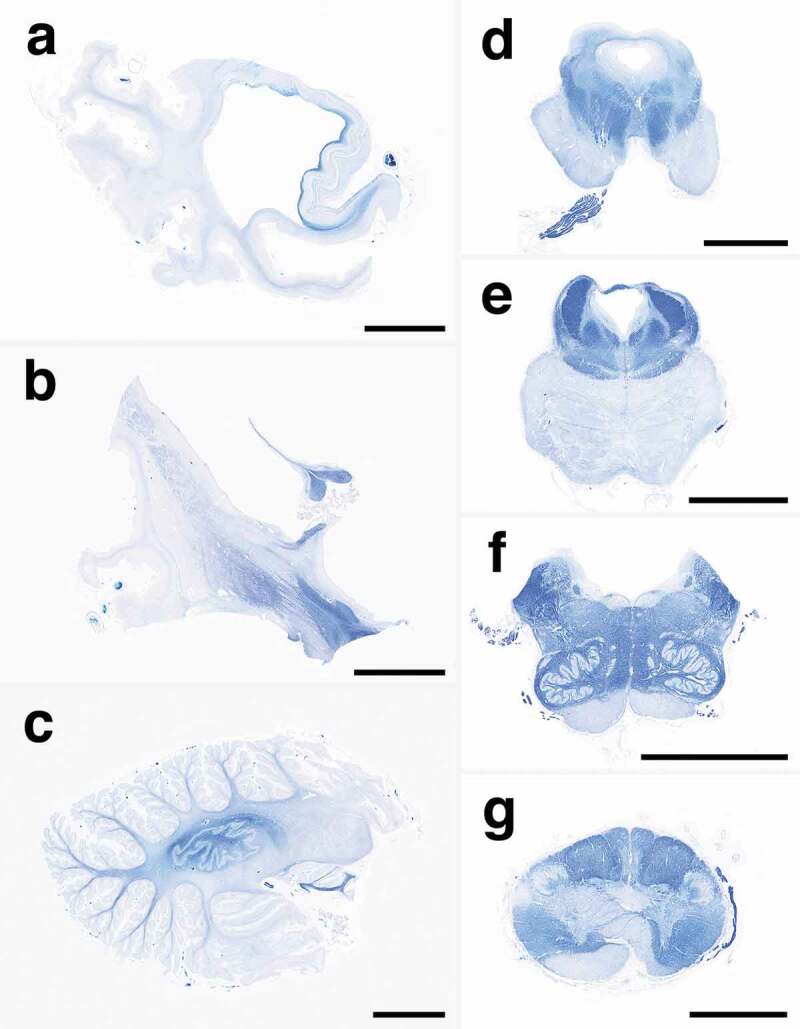
Representative low-power images of myelin with Klüver-Barrera staining. (a) A whole mount of the temporal lobe shows severe atrophy with diffuse myelin pallor of the white matter, suggesting panencephalopathic-type pathology. The hippocampal formation and subiculum are relatively preserved from atrophy with preservation of myelin stainability. (b) The striatum shows severe atrophy, whereas the globus pallidus and subthalamic nucleus are relatively preserved. The internal capsule shows moderate myelin pallor, whereas the body of the fornix is preserved. (c) The cerebellum shows severe atrophy with diffuse myelin pallor, but the dentate nucleus is relatively preserved, and the hilum shows relatively preserved myelin stainability. (d) The midbrain shows severe myelin pallor of the cerebral peduncle, including the frontopontine tract, pyramidal tract, and parietotemporopontine tract. The red nucleus, medial lemniscus, and medial longitudinal fasciculus are preserved from myelin pallor. The oculomotor nerve root is well preserved. (e) The pons shows atrophy of the base with diffuse myelin pallor, including the longitudinal pontine fasciculi and transverse pontine fibres. The tegmentum is relatively preserved from atrophy. The superior cerebellar peduncle, medial longitudinal fasciculus, central tegmental tract, and medial lemniscus are well preserved. (f) The medulla oblongata shows severe atrophy of the pyramid with severe myelin pallor. The tegmentum, medial longitudinal fasciculus, medial lemniscus, and inferior olivary nucleus are well preserved. (g) The upper cervical cord shows severe pyramidal tract degeneration. The posterior column is well preserved. Scale bars: a-f: 10 mm; g: 5 mm

The patient’s brain weighed 960 g before fixation. Macroscopically, severe general atrophy was observed in the cerebrum (Figure 1a). Coronal sections of the cerebrum revealed severe atrophy of the neocortex, striatum, and medial thalamus, and the white matter also showed severe atrophy (Figures 1b, Figures 2a, Figures 2b). In contrast, the globus pallidus, lateral thalamus, amygdala, hippocampal formation, and subiculum were relatively preserved from atrophy. Sagittal sections of the cerebellum revealed atrophy of the cortex and white matter, but the dentate nucleus was relatively preserved (Figure 2c). Axial sections of the brainstem and upper cervical cord showed atrophy of the pontine base and pyramid of the medulla oblongata (Figure 2d–f). Depigmentation of the substantia nigra and locus coeruleus was not apparent. Olivary hypertrophy was not observed.

### Microscopic findings

**Figure 3.**
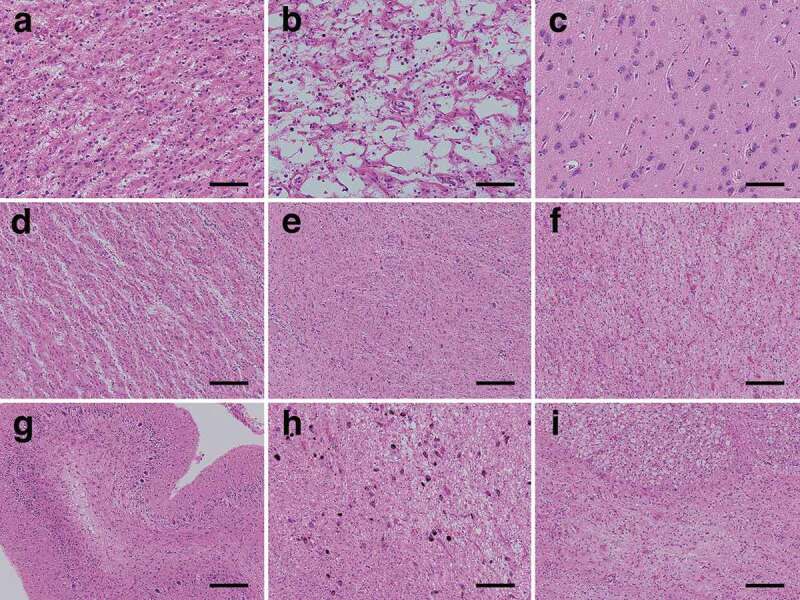
Representative microscopic findings of haematoxylin and eosin-stained specimens. (a) The neuropil shows severe rarefaction, and severe neuronal loss with fibrous gliosis is recognized. Achromatic neurons (inflated neurons) are scattered. Hypertrophic astrocytosis is apparent, and many macrophages are recognized (superior temporal gyrus). (b) Characteristic large cystic cavitation is observed. Macrophages are recognized in the cavities. Hypertrophic astrocytosis is no longer remarkable (parastriate area). (c) Mild vacuolization without apparent gliosis is observed in the subiculum. (d) The putamen shows severe rarefaction and severe neuronal loss with fibrous gliosis. (e) The globus pallidus is relatively preserved with very mild spongiform changes and gliosis. (f) The cerebral white matter shows tissue rarefaction, numerous foamy macrophages, and hypertrophic astrocytosis (occipital lobe). (g) The cerebellar molecular layer shows severe atrophy, and the granule cell layer shows severe neuronal loss, whereas the Purkinje cell layer is relatively preserved from neuronal loss. (h) The substantia nigra shows moderate neuronal loss with gliosis and severe tissue rarefaction. (i) The pontine nucleus shows severe neuronal loss with gliosis. The longitudinal pontine fasciculus shows numerous macrophages. Scale bars: a-c: 100 μm; d-i: 200 μm

The cerebral neocortex showed extensive devastating lesions (status spongiosus), and typical spongiform changes could no longer be observed; the neuropil showed severe rarefaction with hypertrophic astrocytosis, and severe neuronal loss was observed (Figure 3a). Cortical laminar structures were unidentifiable. A characteristic large cavity formation was partially recognized, particularly in the parietal lobe (Figure 3b). In the limbic system, typical spongiform changes with fine vacuolization and without apparent gliosis were observed in the hippocampal formation and subiculum (Figure 3c). The amygdala and Meynert nucleus showed apparent spongiform changes with hypertrophic astrocytosis and mild neuronal loss, whereas the parahippocampal gyrus and insular cortex showed severe degeneration with severe neuronal loss, similar to that of the cerebral neocortex. With regard to the subcortical grey matter, the striatum (Figure 3d) and medial thalamus also showed severe involvement similar to that of the cerebral neocortex, whereas the globus pallidus (Figure 3e), lateral thalamus, subthalamic nucleus, nucleus accumbens, and hypothalamus were relatively preserved with mild spongiform changes and gliosis. In the cerebral white matter, extensive myelin pallor with tissue rarefaction, numerous foamy macrophages, emperipolesis, and hypertrophic astrocytosis were recognized (Figure 3f). The internal capsule was preserved to a slightly greater extent than the surrounding white matter. In the cerebellum, the molecular layer showed severe atrophy (Figure 3g). The granule cell layer showed severe neuronal loss, whereas the Purkinje cell layer was relatively preserved. The white matter of the cerebellum showed severe myelin pallor with tissue rarefaction, whereas the dentate nucleus was relatively preserved. In the brainstem, severe myelin pallor of the cerebral peduncle and conspicuous pyramidal tract degeneration were observed. The substantia nigra showed moderate neuronal loss with gliosis and tissue rarefaction (Figure 3h). Moderate neuronal loss was observed in the quadrigeminal body, and it was severe in the pontine nucleus (Figure 3i). Other brainstem nuclei, including the red nucleus, locus ceruleus, facial nerve nucleus, hypoglossal nerve nucleus, dorsal nucleus of the vagal nerve, and inferior olivary nucleus, were essentially preserved.

**Figure 4.**
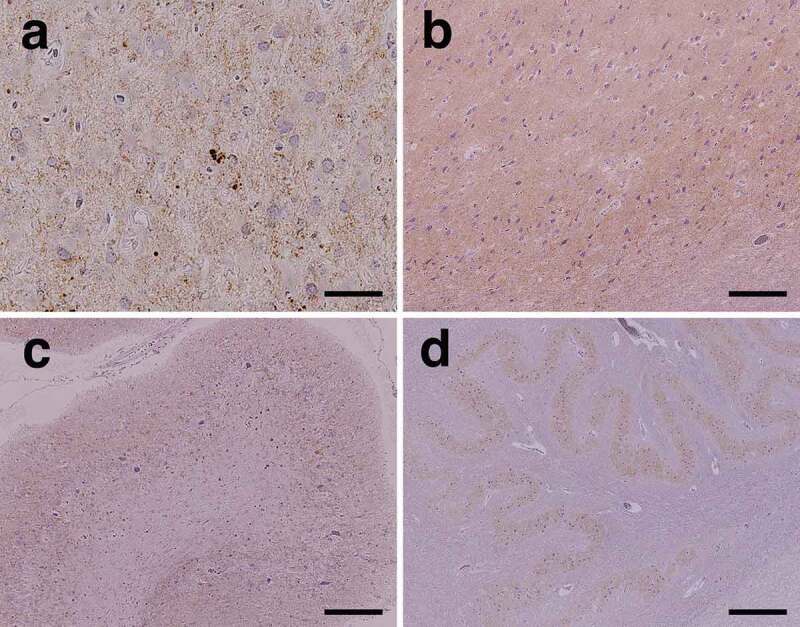
Representative microscopic findings of anti-prion protein (PrP) immunostaining. (a) Diffuse fine granular PrP deposition (synaptic-type) is recognized in the cerebral neocortex. Small plaque-like deposition is scattered (middle temporal gyrus). (b) In the limbic system, diffuse synaptic-type PrP deposition is observed (subiculum). (c) In the cerebellar cortex, PrP deposition is extensively observed in the molecular and granule cell layers. (d) PrP deposition is observed in the inferior olivary nucleus. Scale bars: a: 50 μm; b, c: 200 μm; d: 500 μm

PrP immunostaining revealed extensive diffuse synaptic-type PrP deposition in the grey matter, including the cerebral neocortex (Figure 4a), limbic system (Figure 4b), basal ganglia, and thalamus. Small plaque-like deposition was also observed mainly in the deeper cerebral neocortical layer, whereas perivacuolar-type PrP deposition was not observed. In the cerebellum, diffuse synaptic-type PrP deposition was observed in the molecular and granule cell layers (Figure 4c), and some was observed in the dentate nucleus. In the brainstem, synaptic-type PrP deposition was observed in the substantia nigra, quadrigeminal body, pontine nucleus, inferior olivary nucleus (Figure 4d), dorsal column nuclei, and posterior horn of the upper cervical cord. In the white matter of the brain, PrP deposition was not apparent.

The ageing pathology was essentially mild. A few neurofibrillary tangles (NFTs) were observed using Gallyas-Braak silver staining and AT-8 immunostaining, and the distribution corresponded to Braak stage I [7,8]. Senile plaques were not observed using Gallyas-Braak silver staining and anti-amyloid β immunostaining. No argyrophilic grains were present. Lewy bodies and α-synuclein pathology were absent, as revealed by anti-α-synuclein immunostaining. No apparent vascular lesions were observed.

### Western blot analysis of PrP^Sc^

**Figure 5.**
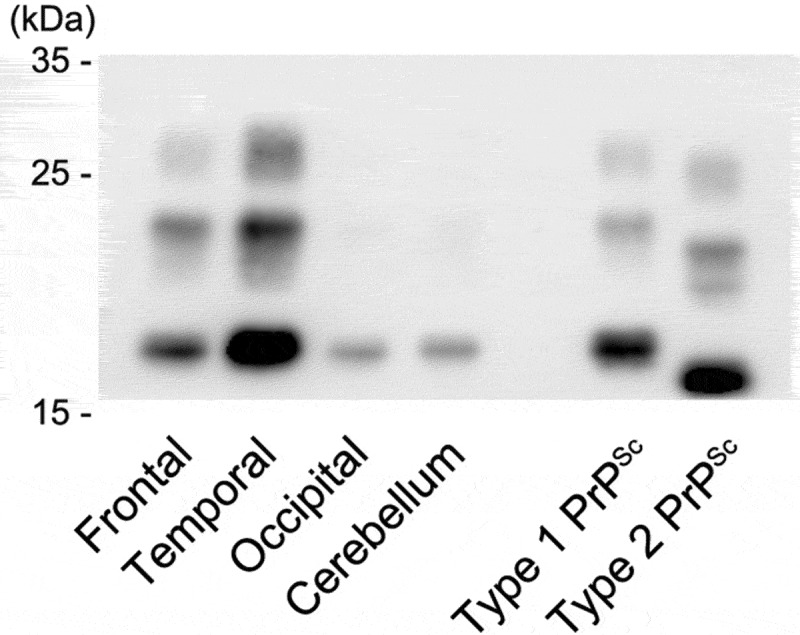
Western blot analysis of proteinase K-resistant prion protein (PrP). The gel mobility of PrP^Sc^ from the present patient was compared with that of other patients with methionine/methionine type 1 (MM1)-type and MM2-type sporadic Creutzfeldt-Jakob disease (sCJD). PrP^Sc^ migrated as three bands, which correspond to the diglycosylated (upper band), monoglycosylated (middle band), and unglycosylated (lower band) forms. The unglycosylated bands appear at approximately 21 kDa in the present case (frontal, temporal, and occipital cortices and cerebellar cortex). The findings indicate type 1 PrP^Sc^. The MM2-type and MM1-type sCJD controls (the right lane and the next lane) show type 2 PrP^Sc^ located at 19 kDa and type 1 PrP^Sc^ located at 21 kDa, respectively

The Western blot analysis using the 3F4 antibody of the present case showed type 1 PrP^Sc^ bands in each examined region (Figure 5). The analysis using the type 1 PrP^Sc^ specific antibody showed apparent positive bands, but that using the type 2 PrP^Sc^ specific antibody showed no positive bands (data not shown). A mixed finding of type 2 PrP^Sc^ was not recognized.

## Discussion

In the present case, the clinical findings observed in the early disease stages were typical of classic-type sCJD [1–3]; however, the patient showed a stable general condition in an akinetic mutism state for more than 2 years. Although survival until the akinetic mutism state is similar between Japanese and North American or European patients with MM1-type sCJD [9], Japanese patients show considerably longer total disease duration [5,9,10]. The extended survival period in Japanese patients results from supportive therapies, including intravenous high-calorie infusions, administration of antibiotics to treat bacterial infections, and appropriate treatments for various complications [9,10]. In terms of symptomatic treatments, tube feeding once the akinetic mutism state is reached is a crucial factor contributing to the prolonged survival of patients with sCJD [10]. More than half of Japanese prion disease cases are supported by tube feeding when the patient has progressed to an akinetic mutism state [9,10]. Nasal tube feeding is selected in most cases because of the concern of prion infection during a more invasive procedure, such as a gastrostomy [10], although Japanese patients with a prion disease who undergo gastrostomy show significantly fewer tube feeding discontinuations due to complications than those who do not [11]. Because prolonged disease duration, similar to that seen in the present case, is rarely seen in classic-type sCJD cases in European and North American countries [1,9], it is suspected that severe and devastating lesions, such as those seen in the present case, are also rarely observed.

In general, the cerebral neocortex is the most severely affected region with respect to MM1-type sCJD pathology, and its severity is associated with total disease duration [2–5], whereas the hippocampal formation and subiculum are consistently spared, regardless of cerebral neocortical pathology or disease duration [1–5,12]. A significant observation of the present case was system degeneration, such as pyramidal tract degeneration, even with unusually prolonged disease duration. According to previous reports, sCJD pathology reflects two distinct pathogeneses [2,3,5]: primary grey matter degeneration due to PrP deposition and secondary degeneration due to primary lesions. Although PrP deposition is extensively observed in the grey matter in MM1-type sCJD cases from the early disease stages, each anatomical region is essentially divided into two types [2,3,5,6,13,14]: 1) hardly affected regions, such as the hippocampal formation, subiculum, globus pallidus, lateral thalamus, inferior olivary nucleus, and cerebellar dentate nucleus, as they carry resistance properties, and 2) readily affected regions, such as the cerebral neocortex, striatum, medial thalamus, and cerebellar cortex, as they are vulnerable to CJD pathological progression due to accumulation of abnormal PrP. Although it remains unclear why the central nervous system regions show either vulnerability or resistance to PrP deposition and pathological progression, it could be connected to neuroembryology [2,3]. In general, the developmentally more recent parts of the brain are found to be considerably involved, whereas older regions tend to exhibit degradation to a lesser extent [2,3]. Alternatively, it may be associated with synaptic function and number in each region [3]. Because this tendency was shown in the present case, distinct preserved regions certainly exist in CJD pathology, even in unusually prolonged cases.

sCJD pathology showing diffuse myelin pallor of the cerebral white matter, such as in the present case, has been classified as panencephalopathic-type, and the pathogenesis of widespread white matter degeneration is suspected due to secondary degeneration caused by extensive cerebral neocortical involvement, particularly with regard to neuronal loss [2–5,15]. CJD with panencephalopathic-type pathology is relatively common in Japan [2,3,5,16], but it is extremely rare in Europe and North America [1,12]. This is because the long-term course is relatively common in CJD cases in Japan, unlike in Europe and North America [2–5,9]. We speculated that if the grey matter, particularly nerve cells, was preserved, the output system from the region could be preserved (as in the present case), such as the central tegmental tract from the red nucleus, the olivocerebellar tract from the inferior olivary nucleus, the superior cerebellar peduncle from the dentate nucleus, and the fornix from the hippocampal formation. Similarly, ascending axonal fibres from the spinal cord or more peripheral parts were essentially preserved in the present patient, such as the lateral and medial lemniscus and posterior column.

The large cavity formation observed in the present case is not common in CJD pathology but is occasionally observed in the cerebral neocortex of CJD cases with extremely long disease duration [2–4]. The characteristic cavitation is similar to the laminar necrosis of the cerebral cortex observed in hypoxic encephalopathy [2,4]. Because ischaemic episodes were not recognized in the present case, and the hippocampus, which is vulnerable to ischaemia, was relatively preserved, we believe that the large cavities were not caused by ischaemic episodes or cerebral blood insufficiency but by the terminal pathology of CJD. We also consider that the term ‘large cavity formation’ must not be confused with ‘spongiform change,’ denoting round vacuoles with clear boundaries, or with ‘large confluent vacuoles,’ denoting large vacuoles in small coalescing groups in the neuropil of the pathologic observation of CJD, and it should be distinguished appropriately.

Although the present patient died due to central respiratory failure, we could not identify the responsible pathological lesion. However, we estimated the cause of death to be related to brainstem pathology, particularly PrP deposition, in the respiratory centre of the medulla oblongata. The formation and accumulation of abnormal PrP is considered a primary event in the early stages of sCJD [2–4,13]; however, the mechanism related to neurodegeneration, including neuronal dysfunction, remains unknown. In our previous observations, widespread PrP deposition in the brainstem grey matter was consistently observed, regardless of disease duration, at least in MM1-type sCJD [2,3,6]; the pattern of distribution was relatively uniform among patients. Interestingly, PrP deposition in the brainstem grey matter was not associated with neuronal loss until the prolonged stage, except for some regions, such as the pontine nucleus [2,3,6]. Furthermore, including, but not limited to, the present case, central respiratory failure (such as Cheyne-Stokes respiration and Biot’s respiration) is an important cause of death in sCJD [6,17]. Based on these observations, it is considered that MM1-type sCJD has a limited survival period because of the appearance of central respiratory failure, even if active symptomatic treatment is provided.

In conclusion, CJD pathology shows system degeneration even in cases with prolonged disease duration; the preserved regions are apparently retained, and not all regions are affected by CJD pathology.

## Patient and methods

### Clinical summary

**Figure 6.**
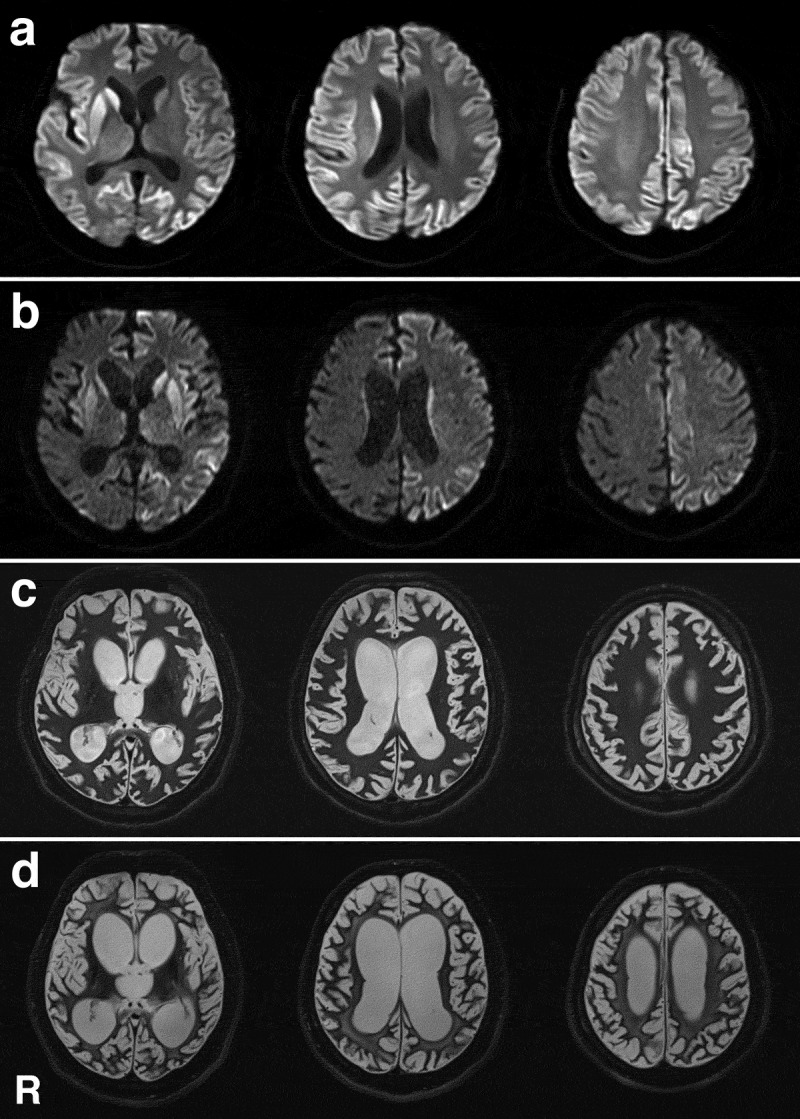
Magnetic resonance images of the brain. (a) Diffusion-weighted images (DWI) obtained 2 weeks after symptom onset show extensive hyperintense regions in the cerebral cortical ribbon and striatum, particularly on the right side. (b) DWI obtained 4 months after symptom onset shows extensive hyperintense regions of the cerebral cortex and striatum, particularly on the left side. Brain atrophy is mildly progressed. (c) T2-weighted images obtained 12 months after symptom onset show general cerebral atrophy and lateral ventricular dilatation. White matter degeneration is still not apparent. (d) T2-weighted images obtained 24 months after symptom onset show severe cerebral atrophy and lateral ventricular dilatation. White matter degeneration is recognized. R, right side

A previously healthy, 48-year-old, Japanese man presented with abnormal behaviour and gait disturbances that developed over the course of a few days. One week after symptom onset, he was admitted to a mental hospital because he loudly spoke nonsensical speech and showed psychiatric symptoms such as restlessness and agitation. In the Department of Psychiatry, he was initially diagnosed with hysteria. His cognitive dysfunction rapidly progressed, and conversation became impossible 2 weeks after symptom onset. Magnetic resonance imaging (MRI) revealed hyperintense regions in the bilateral cerebral cortices and striatum on diffusion-weighted imaging (Figure 6a). Because CJD was clinically suspected based on the clinical and MRI findings at this stage, he was transferred to the Department of Neurology. He had no evidence of iatrogenic CJD aetiology and no family history of prion disease. Neurological examination revealed a startle reaction and left side-dominant myoclonus of his extremities. No apparent muscle weakness was observed, but the patient could not walk due to ataxia. Semi-quantitative analysis revealed positive 14-3-3 proteins and total tau proteins in the cerebrospinal fluid (CSF). A CSF real-time quaking-induced conversion test result was also positive. EEG showed a diffuse slow basic pattern with PSWCs. Genomic analysis of PrP gene-extracted DNA from peripheral blood lymphocytes was performed after obtaining informed consent from the patient’s family members. The analysis revealed no mutations in methionine homozygosity at codon 129 and glutamic acid homozygosity at codon 219. Approximately 2 months after symptom onset, the patient reached an akinetic mutism state, and oral intake became difficult. A gastrostomy was performed per the patient’s family’s request. MRI was performed several times during the disease course and showed progressive brain atrophy and white matter degeneration (Figure 6b–d). Thereafter, he maintained a stable general condition with tube feeding for more than 2 years, but 30 months after symptom onset, he died of central respiratory failure. Tracheotomy and mechanical ventilation were not performed. An autopsy was performed with informed consent from the patient’s family. No apparent sleep disorder, autonomic failure, muscle fasciculations, or sensory symptoms were apparent during the disease course.

### Neuropathological examination

The brain was fixed in 20% neutral buffered formalin for 4 weeks. After brain cutting and trimming, tissue blocks were immersed in 95% formic acid for 1 h to inactivate prion infectivity. Five-micrometre sections were sliced using a microtome, then they were mounted, deparaffinized, dehydrated, and stained. For routine neuropathological examinations, the sections were subjected to haematoxylin and eosin (HE), Klüver-Barrera, and Gallyas-Braak silver staining.

Immunohistochemical analyses of selected sections were performed using anti-PrP antibodies (3F4; Dako, Glostrup, Denmark; mouse monoclonal, diluted 1:100). The immunostaining protocol for PrP detection used a hydrolytic autoclaving procedure, as previously described [5,6]. Immunostaining with anti-Aβ (4G8; Signet, Dedham, MA, USA; mouse monoclonal, diluted 1:2,000), anti-hyperphosphorylated tau (AT-8; Innogenetics, Ghent, Belgium; mouse monoclonal, diluted 1:1,000), and anti-phosphorylated α-synuclein (pSyn#64; Wako Pure Chemical Industries, Osaka, Japan; mouse monoclonal, 1:3,000) was also performed. In each immunostaining procedure, primary antibody binding was detected using the envision-amplified visualization method (EnVision Plus kit; Dako, Glostrup, Denmark). Peroxidase-conjugated streptavidin was visualized with 3,3-diaminobenzidine (Wako Pure Chemical Industries, Osaka, Japan) as the final chromogen. Immunostained sections were lightly counterstained with Mayer’s haematoxylin.

### Western blot analysis of PrP^Sc^

The four cryopreserved brain regions (the frontal, temporal, and occipital cortices and the cerebellar cortex [right side only]), which were snap frozen at autopsy and stored at −80°C prior to use, were homogenized, and Western blot analysis of proteinase K-resistant PrP was performed using 3F4 antibodies, as previously described [5,6]. As positive controls, cryopreserved samples from the cerebral frontal cortices of sCJD cases with the MM1-type and MM2-thalamic-type were also prepared. We performed analysis of each sample corresponding to 0.5 mg of brain tissue (wet weight). PrP^Sc^ typing was performed according to the sCJD classification system proposed by Parchi et al. [1]. Tohoku-1 and Tohoku-2 (type 1 PrP^Sc^ and type 2 PrP^Sc^ specific antibodies, respectively) were also used for confirmation of the typing [18].
